# Reliability of accelerometric measurement of physical activity in older adults-the benefit of using the trimmed sum

**DOI:** 10.1007/s11556-012-0100-x

**Published:** 2012-04-22

**Authors:** Ulrike Sonja Trampisch, Petra Platen, Matthias Trampisch, Anna Moschny, Ulrich Thiem, Timo Hinrichs

**Affiliations:** 1 Department of Sports Medicine and Sports Nutrition, Ruhr-University Bochum, 44801 Bochum, Germany; 2 Department of Medical Informatics, Biometry and Epidemiology, Ruhr-University Bochum, 44801 Bochum, Germany

**Keywords:** Aged, Reproducibility of results, Activities of daily living, Bias (epidemiology)

## Abstract

There is general consensus that physical activity is important for preserving functional capacities of older adults and positively influencing quality of life. While accelerometry is widely accepted and applied to assess physical activity in studies, several problems with this method remain (e.g., low retest reliability, measurement errors). The aim of this study was to test the intra-instrumental retest reliability of a wrist-worn accelerometer in a 3-day measurement of physical activity in older adults and to compare different estimators. A sample of 123 older adults (76.5 ± 5.1 years, 59 % female) wore a uniaxial accelerometer continuously for 1 week. The data were split into two repeated measurement values (week set) of 3 days each. The sum, the 80–99th quantiles and the 80–99th trimmed sums were built for each week set. Retest reliability was assessed for each estimator and graphically demonstrated by Bland–Altman plots. The intraclass correlation of the retest reliability ranged from 0.22 to 0.91. Retest reliability increases when a more robust estimator than the overall sum is used. Therefore, the trimmed sum can be recommended as a conservative estimate of the physical activity level of older adults.

## Introduction

There is general consensus that physical activity is important for preserving functional capacities of older adults and for positively influencing quality of life [7, 12]. To measure physical activity in studies, a variety of direct (e.g., pedometer) or indirect (e.g., questionnaires) methods is used [18, 20]. The measurement methods differ with regard to their quality, criteria validity and retest reliability, costs and acceptance by study participants, and depend closely on the feasibility within the study design. At present, no gold standard for the assessment of physical activity has been established [18, 28].

Among direct methods to measure physical activity, accelerometry is accepted and widely applied. An accelerometer is worn on the body (e.g., at the hip, ankle, or wrist) measuring acceleration in up to three dimensions. In so doing, information on frequency, intensity, and duration of an individual’s physical activity is collected, expressed in “counts per minute” (CPM). It is assumed that the amount of CPM is associated with the intensity of physical activity [5, 14]. To represent the average physical activity of an individual, a minimum of 3-day measurement is suggested [28, 29].

Despite the widespread use, direct measurement of physical activity using an accelerometer remains challenging [18, 31]. There is-for example-no consensus on the type of accelerometer to use [1, 18, 28], nor is there agreement as to the part of the body on which it should be worn [9], just recommendations for different target groups, e.g., for older adults exist [4, 6, 16, 21]. Older adults frequently perform physical activity with light to moderate intensity, such as housekeeping, gardening, or walking for leisure [11]. In order to take these activities into account, some authors recommend the use of a wrist-worn uniaxial accelerometer [4, 6, 16, 21], since movements mainly occur in the upper body and arms (e.g., the wrist-worn “Actiband” AB64 uniaxial accelerometer, Cambridge Neurotechnology Ltd., UK).

Despite the wide use of accelerometry-based measurement of physical activity in all kinds of studies, data on the retest reliability are seldom published [16]. This is true for the uniaxial wrist-worn Actiband accelerometer itself, as well as for other accelerometers in general. The only published data on the retest reliability of the Actiband was found in Rowe et al. [19]. They found a high inter-instrumental retest reliability of two Actibands which were worn simultaneously during a test on a treadmill (ICC = 0.98; 95 % CI: 0.91–0.99). However, the study was performed with ten 10 to 11-year-old boys in a laboratory environment, comparing two different Actibands. Therefore, these results cannot directly be adopted for the measurement of activities of daily life in community-dwelling older adults within a nonlaboratory situation.

Maybe the reason for the limited data of the accelerometer-based measurement of physical activity is partly explained by the disappointing results of the analysis of retest reliability. Usually, the sum of CPM or the mean CPM, collected over a period of a few days and divided by the number of days, [26, 28, 30], is used to express the average amount of physical activity of an individual. The resulting “average counts per day” often show tremendous intra- and inter-individual variability [9]. This variability may be partly explained by outliers of the CPM measured by the accelerometer. Outliers are multiples of reasonable CPM values. These values are defined as measurement errors, since they are clearly due to methodological issues of the manufacture and cannot be achieved by any kind of physical activity. Consequently, using the sum or the mean of CPM which still include the outliers cannot result in high retest reliability. Unfortunately, a standardized recommendation on how to deal with outliers of accelerometry is lacking. Retest reliability might be low due to the outliers which account for the overall sum and not due the general missing possibility of reproducing the results. Orsini et al. [17], for example, defined CPM greater than 20,000 as malfunction of the accelerometer without further explanations on the cut-point they chose. These data were then set as missing and thereby excluded from analyses. Instead of defining a certain cut-point for each accelerometer, we would like to suggest a different approach. In order to enhance the retest reliability, an alternative and more robust estimator that is less sensitive to outliers/measurement errors might be needed. The trimmed (or truncated) sum may be an alternative estimator. The trimmed sum is obtained by omitting a certain percentage of the most extreme observations (e.g., 5 % of the low and 5 % of the high end) and taking the sum of the rest. It is a robust measure of central tendency and is stable against abnormal extreme values (such as measurement errors/outliers), which get “trimmed” away [2]. Using the trimmed sum to express the average amount of physical activity of an individual instead of the overall sum of CPM may result in higher retest reliability.

The aim of the study was therefore to find a more robust estimator in order to account for outliers that occur by using accelerometry to measure physical activity. This more robust estimator will then be used to test the intra-instrumental retest reliability of a wrist-worn accelerometer in a 3-day measurement of physical activity in community-dwelling older adults. We hypothesized that using quantiles and the trimmed sum instead of the overall sum (which includes the outliers) of CPM will decrease the measurement error and increase the retest reliability.

## Methods

The presented study was part of a validation study of a physical activity questionnaire for older adults [23–25]. Participants were recruited via 13 general practitioners in North-Rhine Westphalia, Germany during springtime. All patients, who visited the practice for any reason and fulfilled the inclusion criteria, were asked to become a participant of the study. The inclusion criteria were being 70 years or older, being legally competent and able to cooperate appropriately, and providing written informed consent. The exclusion criteria were life expectancy less than 6 months, being in a wheelchair or bedridden. Recruitment time within one general practitioner practice was 1 week. Body mass index (kg × m^−2^) was computed from measured height and weight on a standard balance scale (Seca 862) and stadiometer (Seca 214, both: Seca, Germany). The study was approved by the Ethics Committee.

A sample of 123 community-dwelling older adults wore the Actiband AB64 uniaxial accelerometer (Cambridge Neurotechnology Ltd., UK) continuously for 1 week (7 days, 24 h per day). The Actiband is a lightweight device (12 g; size, 35 × 15 × 5 mm) that measures and records vertical acceleration with a 1-min epoch. The device is waterproof.

In order to find a more robust estimator than the overall sum of CPM, the retest reliability of selected quantiles and trimmed sums was evaluated and compared to that of the overall sum of CPM. After participants had returned the Actiband to the study center, the complete data of 1 week were afterwards split into two repeated measurement values (week set) of 3 days each. The first set included the CPM data from Monday, Tuesday, and Wednesday; and the second set included the data from Thursday, Friday, and Saturday. Sunday was excluded. Assuming that a maximum of 20 % of the CPM values at the high end of the distribution were due to outliers, these values were discarded by building two datasets each: the 80th, 85th, 90th, 95th and 99th quantiles (Q80–Q99), and the 80, 85, 90, 95, and 99 % trimmed sums (TS80–TS99). The CPM value of the, e.g., Q80 (80th quantile) is the value where 80 % of the CPM values are less or equal to it and 20 % are greater than or equal to the particular CPM value. To build the trimmed sums, only the high end of the CPM values was trimmed, since the low end contained a large number of null values (measured during sleep or physical inactivity), and then summed up. This means that the (1 − *β*) trimmed sum is calculated as

$$ {\text{T}}{{\text{S}}_{1 - \beta }} = { }\mathop \sum \limits_{i = 1}^n {w_i} \cdot {\text{cp}}{{\text{m}}_i} $$

where

$$ {w_i} = \left\{ {\matrix{{*{20}{c}} {1, \frac{i}{n} \leqslant 1 - \beta } \\ {0, \frac{i}{n} > 1 - \beta } \\ } } \right. $$

and 0 ≤ *β* ≤ 1. The parameter *β* defines the percentage of the trimming. For comparison, the special case of the overall sum (*β* = 0, i.e., nothing is trimmed) will also be described.

Over the 1-week measurement period, 10,080 [=7 × (days) × 24 (hours) × 60 (minutes)] single values of CPM were collected per participant. Each original 3-day set consequently consisted of 4,320 [=3 × (days) × 24 (hours) × 60 (minutes)] single values. The retest reliability was assessed by using the intraclass correlation (ICC A, 1) [15].

A Bland–Altman plot was used as a graphic assessment of the agreement of the 2 week sets where the difference between the 2 week sets is plotted against their mean for each subject [3]. The 95 % limits of agreement, estimated by mean difference ±1.96 × standard deviation of the differences, provide an interval within which 95 % of differences between measurements by the 2-week sets are expected to lie.

## Results

### Participants

The anthropometric characteristics of the 123 participants (aged 76.5 ± 5.1 years, 59 % female) of the study are shown in Table 1.

**Table 1:** Characteristics of the study participants in total and broken down by sex

	Total *n* = 123			Female *n* = 73 (59 %)			Male *n* = 50		
	Mean ± SD	Min	Max	Mean ± SD	Min	Max	Mean ± SD	Min	Max
Age (years)	76.5 ± 5.1	70.1	98.6	77.2 ± 5.8	70.1	98.6	75.6 ± 3.7	70.2	85.5
Height (m)	1.65 ± 0.08	1.47	1.86	1.60 ± 0.06	1.47	1.78	1.71 ± 0.06	1.54	1.86
Weight (kg)	81.2 ± 14.5	50.5	114.0	77.3 ± 14.4	50.5	114.0	86.8 ± 12.7	55.0	111.0
Body mass index (kg × m²)	29.8 ± 4.4	20.5	44.2	30.1 ± 4.9	20.5	44.2	29.5 ± 3.6	22.9	35.9
Sum CPM over 7 days	619,084^a^	430,340^b^	820,977^c^	692,898^a^	426,588^b^	875,139^c^	594,925^a^	429,553^b^	708,919^c^

*n* number of participants, *SD* standard deviation, *min* minimum, *max* maximum, *m* meter, *kg* kilogram

^a^median

^b^25 % quantile

^c^75 % quantile

### CPM

**Fig. 1:**
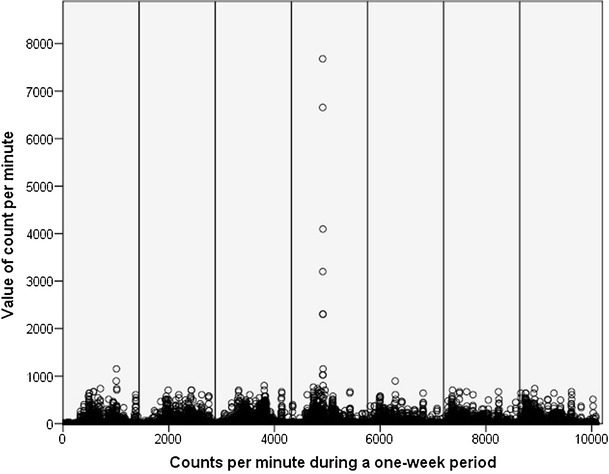
Listed count per minute during a 1-week period (10,080 in total) and each value of the corresponding count per minute including five unreasonable high values (measurement errors). The *solid vertical lines* divide the 7 days of measurement from 1 day to another

The assessment of physical activity of older adults in this study showed measurement errors (outliers). Figure 1 shows the 1-week measurement of a participant. This activity profile mainly shows CPM in between 0 (inactive) and 1,000, whereas five single values clearly exceed the usual level. These values can be defined as outliers. This occurrence was similar in physical activity profiles of other participants who were randomly chosen.

### Retest reliability

**Fig 2:**
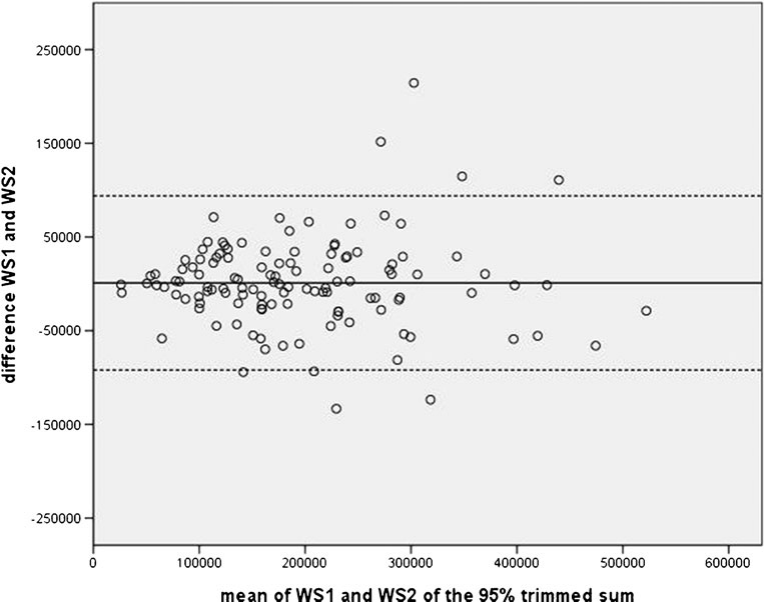
A 95 % trimmed sum of counts per minute: difference (week set 1–week set 2) versus average of values measured in week set 1 and week set 2 with 95 % limits of agreement (mean, ±1.96 × standard deviation). *WS1* week set 1 consisting of Monday, Tuesday, Wednesday; *WS2* week set 2 consisting of Thursday, Friday, Saturday; *CPM* counts per minute, *solid horizontal line* average of differences of WS1 and WS2, *dashed horizontal lines* 95 % limits of agreement (mean difference, ±1.96 × standard deviation)

The results of the retest reliability are summarized in Table 2. The ICC of the retest reliability of the overall sum of CPM for the 3-day measurement was ICC = 0.77 (95 % confidence interval [CI]: 0.68–0.83). Using the quantiles, the ICC was between 0.22 (95 % CI: 0.05–0.39) and 0.91 (95 % CI: 0.87–0.93). For the trimmed sum, the ICC was between 0.82 (95 % CI: 0.75–0.87) and 0.85 (95 % CI: 0.79–0.89). The differences in the CPM from week set 1 and week set 2 show only moderate variations in the Bland–Altman plots using TS95 (Fig. 2).

**Table 2:** Counts per minute of the sum, the quantile, and the trimmed sum including intraclass correlation

	WS1 mean ± SD	Diff (WS1 – WS2) ± SD	ICC (95 % CI)
	WS2 mean ± SD		
Q80	119 ± 66	4 ± 30	0.89 (0.85; 0.92)
	115 ± 62		
Q85	158 ± 79	5 ± 33	0.90 (0.87; 0.93)
	153 ± 74		
Q90	212 ± 96	6 ± 39	0.91 (0.87; 0.93)
	205 ± 87		
Q95	303 ± 125	9 ± 57	0.88 (0.83; 0.91)
	294 ± 107		
Q99	523 ± 221	−3 ± 372	0.22 (0.05; 0.39)
	525 ± 359		
TS80	69,773 ± 49,938	−1,508 ± 28,406	0.84 (0.78; 0.88)
	71,280 ± 50,078		
TS85	98,674 ± 64,107	−2,304 ± 35,615	0.85 (0.79; 0.89)
	100,978 ± 63,797		
TS90	136,415 ± 80,600	−3,165 ± 44,821	0.84 (0.78; 0.89)
	139,579 ± 79,010		
TS95	187,115 ± 99,191	−5,156 ± 55,106	0.84 (0.78; 0.89)
	192,271 ± 97,347		
TS99	248,356 ± 119,996	−6,514 ± 70,062	0.82 (0.75; 0.87)
	254,871 ± 114,305		
Overall sum	273,470 ± 127,960	−9,720 ± 85,596	0.77 (0.68; 0.83)
	283,189 ± 123,043		

*WS1* week set 1, *WS2* week set 2, *SD* standard deviation, *diff* difference, *ICC* intraclass correlation, *95 % CI* 95 % confidence interval

## Discussion

In order to measure physical activity within studies, accelerometry is a measurement technique which is widely applied. But this method is still afflicted with some peculiarities, e.g., measurement errors. In order to enhance the quality of data, we proposed to use more robust estimators such as quantiles or trimmed sums as a summary measure of physical activity instead of the overall sum. These estimators were then analyzed in terms of retest reliability.

As assumed, both quantiles and trimmed sums CPM increased the retest reliability compared to the overall sum. This was true irrespective of the chosen cut off point (e.g., 80 or 95 %) above which the values were omitted (trimmed sum). We clearly showed that the retest reliability increases when using an estimator such as the TS95. We prefer to use the trimmed sum rather than a certain quantile. When using a quantile to describe the physical activity, it remains unclear whether this CPM value at a predefined quantile was achieved due to one intensive physical activity (e.g., running quickly to get to the bus once during the 3-day measurement period) irrespective of the remaining physical activity or if the person has a high average level of overall physical activity in general. In contrast to the quantiles, the trimmed sum accounts for this, and the average physical activity remains in the estimator. The trimmed sum allows a predefined (or estimated) error level *β* of the used device to be selected, and then yields a single number which reflects all activities. Additionally, the trimmed sum is proportional to the weighted/truncated mean, which allows the calculation of average activity [13], e.g.

$$ {\text{average activity}} = \frac{1}{{\mathop \sum \nolimits_{i = 1}^n {w_i}}} \cdot {\text{T}}{{\text{S}}_{1 - \beta }} $$

Summing up, the advantage of the trimmed sum is that it includes both the average level of activity and more intense effort, whereas a quantile would only measure the peak of more intense activities. Even though the TS95 did not show the highest retest reliability, it seems to be the most conservative estimator of the physical activity. Virtually all measurement errors are eliminated, while all background level and intensive activities are still included. Summing up, the TS95 appears to be an appropriate measurement value for demonstrating the physical activity level of older adults.

Besides the introduced recommendation on dealing with outliers, we would like to compare the retest reliability of our study to others implemented on the same target group. With special regard to other manufacturers of wrist-worn accelerometers, Gao and Tsang [8] found a high retest reliability of the mean CPM in their study on 3-day measurements on 12 people (ICC = 0.98; 95 % CI: 0.93–0.99). These older participants, aged 79.8 ± 11.2 years, wore the uniaxial Actiwatch accelerometer (Mini Mitter Co., Inc., Bend, OR, USA) on the wrist for the same 3 days during two consecutive weeks. Harris et al. [10] found a comparably high agreement of a 7-day measurement with the Actigraph uniaxial accelerometer (GT1M; Manufacturing Technology Inc), worn on the hip by 20 participants (mean age about 74 years) with Pearson’s *r* = 0.87. They repeated the measurement after 2 months.

These two studies both have a more laboratory character compared to our study, since the measurement was repeated on the same days of two consecutive weeks or a whole week after 2 months. In contrast, we took the same week and split it into half (Sunday excluded). With our results on retest reliability, we were able to demonstrate that it is not compulsory to measure physical activity on the same half of the week. Moreover, we also included Saturdays in our analysis, even though activities on a Saturday may be different to the rest of the week. We assumed that participants would be less active on Saturdays compared to Monday to Friday. If this was true, our results on retest reliability would be negatively influenced and would have been even higher, if we had not included Saturdays in the analysis. These two differences in the study design might have relevance for further studies.

## Perspectives

Accelerometry is a method widely applied to assess physical activity in studies [17, 22, 27] and it will often be used in the future. Our recommendation of using the trimmed sum (e.g., 95 %) as a conservative estimate of the physical activity level of older adults would increase the quality of data by controlling for measurement errors.
